# Evidence of Zika Virus Infection in Pigs and Mosquitoes, Mexico

**DOI:** 10.3201/eid2702.201452

**Published:** 2021-02

**Authors:** Daniel Nunez-Avellaneda, Rosa Carmina Cetina-Trejo, Emily Zamudio-Moreno, Carlos Baak-Baak, Nohemi Cigarroa-Toledo, Guadalupe Reyes-Solis, Antonio Ortega-Pacheco, Gerardo Suzán, Chandra Tandugu, Julián E. García-Rejón, Bradley J. Blitvich, Carlos Machain-Williams

**Affiliations:** Universidad Autonoma de Yucatan, Merida, Mexico (D. Nunez-Avellaneda, R. Carmina Cetina-Trejo, E. Zamudio-Moreno, C. Baak-Baak, N. Cigarroa-Toledo, G. Reyes-Solis, J.E. García-Rejón, C. Machain-Williams);; Iowa State University, Ames, Iowa, USA (D. Nunez-Avellaneda, C. Tandugu, B.J. Blitvich);; Universidad Autónoma de Yucatán, Xmatkuil, Mexico; (A. Ortega-Pacheco);; Universidad Nacional Autónoma de México, Mexico City, Mexico (G. Suzán)

**Keywords:** flavivirus, Zika virus, Mexico, pigs, mosquitos, Culex, viruses, zoonoses, vector-borne diseases, mosquito-borne diseases

## Abstract

Evidence suggests that pigs seroconvert after experimental exposure to Zika virus and are potential sentinels. We demonstrate that pigs are also susceptible to natural Zika virus infection, shown by the presence of antibodies in domestic pigs in Yucatan, Mexico. Zika virus RNA was detected in 5 species of mosquitoes collected inside pigpens.

Pigs are susceptible to experimental Zika virus infection (*1*–*4*), but evidence of natural infection is lacking. Microcephaly has occurred in fetal piglets after in utero inoculation, and neurologic disease has occurred in neonates after intracranial inoculation, suggesting that pigs are a suitable animal model for the study of Zika virus. Three-month-old pigs exposed to Zika virus through subcutaneous and intradermal injection produce antibodies but not viremias, indicating that pigs could be suitable sentinels. We performed a serologic investigation in the state of Yucatan, Mexico, to determine whether pigs are susceptible to natural Zika virus infection. Mosquitoes temporally and spatially associated with the pigs were tested for evidence of Zika virus infection to increase our understanding of the vector range of the virus.

## The Study

Pigs and mosquitoes were sampled at 4 sites. One site was a commercial farm in Xmatkuil, a suburb 16 km south of Merida, the largest city in Yucatan. The site contained a herd of Yucatan black hairless pigs and a commercial genetic line of breeding pigs. The other sites were Mayan villages to the east and southeast of Merida: Tzucacab (148 km southeast), Valladolid (159 km east), and Xkalakdzonot (155 km southeast). Each village maintained herds of Yucatan black hairless pigs as a food source for residents. We visited each site 1–3 times during 2018 and 2019, and no pigs were sampled more than once. An unusually high number of porcine fetal deaths occurred in Xmatkuil and Xkalakdzonot several weeks before our initial visits. The stillborn pigs displayed signs of mummification but no apparent neurologic malformations, according to their owners. During each visit, we searched human-made structures and vegetation for resting mosquitoes, which were collected by manual aspiration.

Serum samples were assayed by plaque-reduction neutralization test (PRNT) using dengue virus (DENV) serotype 1 (strain Hawaii), DENV serotype 2 (strain NGC), DENV serotype 3 (strain H-87), DENV serotype 4 (strain 241), Ilheus virus (original strain), St. Louis encephalitis virus (strain TBH-28), West Nile virus (strain NY99–35261–11), and Zika virus (strain PRVABC59). Serum specimens were initially screened at a dilution of 1:20 by using Zika virus. Positive samples were further diluted, then assayed using all 8 viruses. Titers were expressed as the reciprocal of serum dilutions yielding >90% reduction in the number of plaques (PRNT_90_). For etiologic diagnosis, the PRNT_90_ antibody titer to the respective virus was required to be >4-fold that of other flaviviruses tested.

Mosquitoes were transported alive to the arbovirus laboratory at the Universidad Autonoma de Yucatan and sorted into pools of <50 according to species, sex, date, study site, and location within the study site. Mosquitoes were transported in RNAlater (Sigma-Aldrich, https://www.sigmaaldrich.com) to Iowa State University, then homogenized by using mortars and pestles. Total RNA was extracted by using Trizol Reagent (ThermoFisher Scientific, https://www.thermofisher.com) and tested for Zika virus RNA by using reverse transcription PCR and Sanger sequencing using primers that amplify a 667-nt region of the envelope protein gene.

Serum specimens were collected from 297 pigs (20 from Tzucacab, 73 from Valladolid, 74 from Xkalakdzonot, and 130 from Xmatkuil). Thirty-eight (12.8%) pigs were positive for flavivirus-specific antibodies. Thirteen (4.8%) pigs were seropositive for Zika virus, 1 (0.3%) pig was seropositive for West Nile virus, and 24 (8.1%) pigs had antibodies to an undetermined flavivirus. Zika virus PRNT_90_ titers ranged from 40 to 320 (Table 1). Eleven pigs seropositive for Zika virus were from Xmatkuil, and 1 each was from Tzucacab and Valladolid.

**Table 1:** Plaque-reduction neutralization test data for pigs seropositive for Zika virus, Yucatan, Mexico, 2018–2019*

Serum ID	Sample date†	Age category	Virus and PRNT_90_ titer							
			DENV-1	DENV-2	DENV-3	DENV-4	ILHV	SLEV	WNV	Zika virus
XM-278-J‡	2018 Apr	J	–	20	–	–	–	–	–	320
XM-285-J	2018 Apr	J	–	40	–	20	–	–	–	160	
VA-265-A	2018 Jun	A	–	–	–	–	–	–	–	80	
XM-O2A-J	2018 Jun	J	–	20	20	–	40	20	40	160	
XM-177-J	2018 Jun	J	–	–	–	–	–	–	–	40	
XM-181-J	2018 Jun	J	–	–	20	20	–	–	–	80	
XM-183-S	2018 Jun	S	–	–	–	–	–	–	–	40	
XM-189-J	2018 Jun	J	80	–	20	20	40	–	40	320	
XM-199-J	2018 Jun	J	–	–	–	–	–	–	–	40	
XM-202-J	2018 Jun	J	–	20	20	–	–	–	–	80	
XM-212-J	2018 Jun	J	–	–	–	–	–	–	–	80	
XM-238-J	2018 Jun	J	–	–	–	–	–	–	–	40	
TZ-387-J	2019 Jan	J	–	–	–	–	–	–	–	80	

*A, adult; DENV1, dengue virus type 1; DENV2, dengue virus type 2; DENV3, dengue virus type 3; DENV4, dengue virus type 4; ILHV, Ilheus virus; J, juvenile; PRNT_90_, >90% reduction in the number of plaques on plaque-reduction neutralization test; S, suckling; SLEV, St. Louis encephalitis virus; WNV, West Nile virus; –, <20. †Date (month/year) of serum collection. ‡Prefixes indicate pigs from these areas: TZ, Tzucacab; VA, Valladolid; XM, Xmatkuil.

The entomologic investigation yielded 1,870 mosquitoes of 8 species that were sorted into 190 pools. Of these, 381 mosquitoes were collected inside pigpens, and >50% were engorged (Table 2). Mosquitoes were tested for Zika virus RNA by reverse transcription PCR, and resulting amplification products were analyzed by Sanger sequencing. Five pools, all of which contained >1 engorged mosquito, were positive for Zika virus sequence, and all consisted of mosquitoes collected inside pigpens in Xmatkuil (Genbank accession nos. MT309004–309008). One pool each of the following mosquito species tested positive: *Aedes aegypti, Ae. taeniorhynchus, Culex lactator, Cx. nigripalpus,* and *Cx. thriambus*. All sequences were identical and differed from the positive control, an isolate from the state of Chiapas, Mexico, in 2016 (Genbank accession no. KX446950.2) in 1 nucleotide position, a C→T substitution at genomic position 1893.

**Table 2:** Summary of mosquitoes collected inside pigpens, Yucatan, Mexico, 2018–2019

Study site	Mosquito species	No. collected	No. pools	No. pools positive for Zika virus RNA
Tzucacab	*Aedes aegypti*	58	6	0
	*Ae. taeniorhynchus*	3	1	0
	*Culex quinquefasciatus*	63	7	0
	*Cx. thriambus*	1	1	0
Valladolid	*Ae. aegypti*	32	2	0
	*Ae. cozumelensis*	1	1	0
	*Cx. quinquefasciatus*	45	5	0
Xkalakdzonot	*Ae. aegypti*	46	5	0
	*Ae. cozumelensis*	2	1	0
	*Anopheles albimanus*	6	1	0
	*Cx. lactator*	1	1	0
	*Cx. quinquefasciatus*	60	6	0
Xmatkuil	*Ae. aegypti*	29	4	1
	*Ae. taeniorhynchus*	8	2	1
	*Cx. lactator*	1	1	1
	*Culex nigripalpus*	3	1	1
	*Culex quinquefasciatus*	21	2	0
	*Culex thriambus*	1	1	1

## Conclusions

We detected Zika virus RNA sequence in *Ae. aegypti, Ae. taeniorhynchus, Cx. lactator, Cx. nigripalpus,* and *Cx. thriambus* mosquitoes that were temporally and spatially associated with pigs seropositive for this virus. The role of *Culex* spp. mosquitoes in Zika virus transmission has been debated, but the consensus among the arbovirus community is that they are inefficient vectors (*5*,*6*). *Culex* spp. mosquitoes and Zika virus were first linked after experimental infection studies demonstrated that the *Cx. quinquefasciatus* mosquito is a competent vector of this virus (*7*). Many other studies have shown otherwise, including a study that demonstrated that *Cx*. *quinquefasciatus* mosquitoes in the state of Jalisco, Mexico, were refractory to Zika virus (*5*,*6*,*8*).

Vector competence experiments have also evaluated mosquitoes from >6 other *Culex* spp., although *Cx. lactator, Cx. nigripalpus,* and *Cx. thriambus* mosquitoes are not among them, and none were able to transmit Zika virus (*9*). We add to the small number of studies that have detected Zika virus nucleic acid in field-collected *Culex* spp. mosquitoes (*7*,*10*), but we did not isolate virus or provide evidence of a disseminated infection. We cannot dismiss the possibility that the Zika virus RNA–positive *Culex* spp. mosquitoes had recently fed upon a viremic host but virus replication had not occurred within the mosquito. Therefore, the link between *Culex* spp. mosquitoes and Zika virus remains tenuous. The *Ae. taeniorhynchus* mosquito is also considered an inefficient vector of Zika virus (*11*). In contrast, the *Ae. aegypti* mosquito is the principal urban vector of Zika virus in the Americas (*12*). *Ae. taeniorhynchus* and *Ae. aegypti* mosquitoes are not known to have a strong preference for porcine blood, although 2.4% of engorged *Ae. taeniorhynchus* mosquitoes in the Galapagos Islands had acquired blood from pigs, and the *Cx. nigripalpus* mosquito shifts seasonally to opportunistic feeding behavior (*13*,*14*). Porcine blood has occasionally been detected in *Ae. aegypti* mosquitoes (*15*,*16*).

The mosquito infection rates in our study are high. All Zika virus RNA–positive mosquitoes and most seropositive pigs were sampled at the same site (Xmatkuil) on the same date (June 5, 2018). We speculate that these pigs were infected with Zika virus just before our visit and that some mosquitoes then bit them, without virus disseminating from the midguts of *Culex* spp. mosquitoes. Recent studies have demonstrated that pigs are susceptible to experimental Zika virus infection (*1*–*4*). We provide serologic evidence that pigs are also susceptible to natural Zika virus infection. A high number of stillbirths occurred at 2 study sites before sampling, but none displayed malformations typical of Zika virus infection.

We provide additional evidence that pigs produce neutralizing antibodies upon Zika virus exposure and are potential sentinels. This information will be useful for investigators and public and veterinary health personnel conducting surveillance in Zika virus–endemic areas where pigs are common and usually raised outdoors. One limitation of our study is that pig farmers were not tested for evidence of flavivirus infection. Future studies should investigate whether those persons are at increased risk for Zika disease.
